# DART: Denoising Algorithm based on Relevance network Topology improves molecular pathway activity inference

**DOI:** 10.1186/1471-2105-12-403

**Published:** 2011-10-19

**Authors:** Yan Jiao, Katherine Lawler, Gargi S Patel, Arnie Purushotham, Annette F Jones, Anita Grigoriadis, Andrew Tutt, Tony Ng, Andrew E Teschendorff

**Affiliations:** 1 KCL-UCL Comprehensive Cancer Imaging Center, Guy's Campus, London SE1 1UL, UK; 2 Statistical Genomics Group, Paul O'Gorman Building, UCL Cancer Institute, University College London, 72 Huntley Street, London WC1E 6BT, UK; 3 Richard Dimbleby Department of Cancer Research, Randall Division & Division of Cancer Studies, King's College London, WC2R 2LS, UK; 4 Research Oncology, 3rd Floor, Bermondsey Wing,Guy's Hospital, Great Maze Pond, London, SE1 9RT, UK; 5 Breakthrough Breast Research Unit, Guy's Hospital, King's Health Partners Academic Health Sciences Centres, London, SE1 9RT, UK; 6 Department of Radiology, St Thomas' Hospital, London SE1 7EH, UK

## Abstract

**Background:**

Inferring molecular pathway activity is an important step towards reducing the complexity of genomic data, understanding the heterogeneity in clinical outcome, and obtaining molecular correlates of cancer imaging traits. Increasingly, approaches towards pathway activity inference combine molecular profiles (e.g gene or protein expression) with independent and highly curated structural interaction data (e.g protein interaction networks) or more generally with prior knowledge pathway databases. However, it is unclear how best to use the pathway knowledge information in the context of molecular profiles of any given study.

**Results:**

We present an algorithm called DART (Denoising Algorithm based on Relevance network Topology) which filters out noise before estimating pathway activity. Using simulated and real multidimensional cancer genomic data and by comparing DART to other algorithms which do not assess the relevance of the prior pathway information, we here demonstrate that substantial improvement in pathway activity predictions can be made if prior pathway information is denoised before predictions are made. We also show that genes encoding hubs in expression correlation networks represent more reliable markers of pathway activity. Using the Netpath resource of signalling pathways in the context of breast cancer gene expression data we further demonstrate that DART leads to more robust inferences about pathway activity correlations. Finally, we show that DART identifies a hypothesized association between oestrogen signalling and mammographic density in ER+ breast cancer.

**Conclusions:**

Evaluating the consistency of prior information of pathway databases in molecular tumour profiles may substantially improve the subsequent inference of pathway activity in clinical tumour specimens. This de-noising strategy should be incorporated in approaches which attempt to infer pathway activity from prior pathway models.

## Background

A key goal in cancer genomics is to map out the activation levels of cancer-relevant pathways across clinical tumour specimens [1]. Obtaining pathway activity levels is important for several reasons. First, it reduces the genomic complexity from tens of thousands of features to measurements on only dozens of relevant pathways, thus circumventing the significant problems associated with multiple testing [2]. Second, it represents an important step towards understanding the functional effects of genomic and epigenomic abnormalities in clinical tumours [3]. Third, obtaining molecular pathway correlates of clinical and imaging traits may help improve current prognostic and predictive models as well as provide us with important new biological insights [3-5].

However, obtaining reliable estimates of molecular pathway activity is a challenging endeavour. Various gene expression based approaches have been used to address this problem. Initial methods focused on inferring differential pathway activity between biological conditions using Gene Set Enrichment Analysis methods [6,7]. These methods used prior knowledge pathway databases, but only treated pathways as unstructured lists of genes. Proper systems biology approaches that attempt to infer differential pathway activity by combining highly curated structural networks of molecular interactions (e.g KEGG pathway database) with transcriptional changes on these networks were subsequently developed [8-14]. These systems biology approaches can be distinguished depending on whether the discriminatory genes or gene subnetworks are inferred *de-novo *in relation to a phenotype of interest [9-11,14], or whether the molecular pathway models are given as prior information [12,13]. These latter methods are particularly appropriate in conjunction with prior information pathway resources such as Netpath [15]. It is important to stress again that most of these methods (e.g [9-12,14]) are geared towards measuring differential pathway activity and are thus supervised in the sense that the phenotypic information is used from the outset to infer discriminatory genes or gene subnetworks.

Another set of gene expression based approaches are based on deriving perturbation signatures of activation or inhibition in model cell systems and are based on the assumption that the measured downstream transcriptional consequences of the upstream perturbations constitute faithful representations of upstream pathway activity [1,3,16-18]. By correlating these *in-vitro *perturbation mRNA signatures to a sample gene expression profile one may infer pathway activity in individual samples, for example in tumours where one may wish to know the potential functional impact of a particular oncogenic amplification [1,3].

Mathematically, a perturbation signature has the structure of a gene list with associated weights informing us if a gene in the list is up or downregulated in response to gene/pathway activation. Similarly, the Netpath signatures consist of curated lists of genes reported to be up or downregulated in response to pathway activation, and of genes reported to be implicated in the signal transduction of the pathway [15]. Thus, at an elementary level, all of these pathway signatures can be viewed as gene lists with associated weights which can be interpreted as prior evidence for the genes in the list to be up or downregulated.

A common theme of most of the pathway activity estimation procedures described above is the assumption that all of the prior information relating to the pathway is relevant, or that it is all of equal relevance, in the biological context in which the pathway activity estimates are desired. While one would attempt to minimize differences between the biological contexts, this is often not possible. For instance, an *in-vitro *derived perturbation signature may contain spurious signals which are specific to the cell-culture but which are not relevant in primary tumour material. Similarly, a curated signal transduction pathway model may include information which is not relevant in the biological context of interest. Given that personalised medicine approaches are proposing to use cell-line models to assign patients the appropriate treatment according to the molecular profile of their tumour [1], it is therefore important to develop algorithms which allow the user to objectively quantify the relevance of the prior information (e.g pathway model or perturbation signature) before pathway activity is estimated [5]. Similarly, there is a growing interest in obtaining molecular pathway correlates of imaging traits, such as for example mammographic density in breast cancer [4,19-21]. This also requires careful evaluation of prior pathway models before estimating pathway activity. More generally, it is still unclear how best to combine the prior information in perturbation expression signatures or pathway databases such as Netpath with cancer gene expression profiles.

The purpose of this manuscript is four-fold. First, to highlight the need for denoising prior information in the context of pathway activity estimation. We demonstrate, with explicit examples, that ignoring the denoising step can lead to biologically inconsistent results. Second, we propose an unsupervised algorithm called DART (Denoising Algorithm based on Relevance network Topology) and demonstrate that DART provides substantially improved estimates of pathway activity. Third, we use DART to make an important novel prediction linking estrogen signalling to mammographic density data in ER positive breast cancer. Fourth, we provide an assessment of the Netpath resource information in the context of breast cancer gene expression data.

While an unsupervised algorithm similar to DART was used in our previous work [5], we here provide the detailed methodological comparison of DART with other unsupervised methods that do not attempt to de-noise prior information, demonstrating the viability and critical importance of the denoising step. Finally, we also evaluate DART against a state of the art supervised method, called Condition Responsive Genes (CORG) [11], and show that, despite DART being unsupervised, that it performs similarly to CORG. DART is available as an R-package from *cran.r-project.org*.

## Methods

### Perturbation signatures

We considered three different perturbation signatures, all derived by a perturbation (overexpression or inhibition) affecting a single gene in a cell-line model. Specifically, the perturbation signatures were an *ERBB2 *perturbation signature derived by stably overexpressing *ERBB2 *in an ER+ breast cancer cell line (MCF7) [17], a *MYC *perturbation signature derived using a recombinant adenovirus to overexpress *MYC *in human mammary epithelial cells [1], and finally a *TP53 *perturbation signature derived by inhibition of protein synthesis by cycloheximide (CHX) in a human lung cancer cell-line [22]. *ERBB2 *and *MYC *are well-known oncogenes in a wide range of cancers, including breast cancer [23]. *TP53 *is the tumour suppressor gene which is most frequently inactivated in cancer [23].

### The Netpath resource

The Netpath resource [15] (http://www.netpath.org) is a growing, highly curated, database of important signal transduction pathways relevant to cancer and immunology. At the most elementary level these pathways consist of genes whose coding proteins are implicated in the actual signal transduction pathway as well as downstream genes that have been reported to be up and downregulated in response to pathway stimuli. This list of up and downregulated genes therefore provides a measure of pathway activity, provided these genes are relevant in the given biological context. To ensure that correlations between two different pathway activity levels were not due to trivial overlaps of their downstream transcriptional modules, we always calculated activity inference for each pathway in a given pair by only considering the mutually exclusive gene sets. Of all Netpath signatures, we considered ones which have been documented to play important roles in cancer tumour biology, cancer immunology and tumour progression, specially in breast cancer: a6b4 (alpha-6 beta-4 integrin signalling pathway), AR (Androgen receptor), BCellReceptor, EGFR1 (epidermal growth factor receptor-1), IL1,2,3,4,5,6,7,9 (Interleukin 1,2,3,4,5,6,7,9 signalling pathways), KitReceptor (Kit is a receptor protein tyrosine kinase, which is a receptor for stem cell factor or kit ligand), Notch (Notch proteins are important in lineage specification and stem cell maintenance and aberrant Notch signaling has been linked to a number of malignancies including breast cancer), RANKL (Receptor activator of nuclear factor-kappa B ligand (RANKL) is a member of tumor necrosis factor (TNF) superfamily), TCellReceptor, TGFB (transforming growth factor beta signalling) and TNFA (the Tumor Necrosis Factor alpha is a proinflammatory cytokine belonging to the TNF superfamily). Because of the documented role of these pathways in breast cancer, these were used in the context of primary breast cancer gene expression data sets.

### Gene expression data sets used

We used a total of six breast cancer gene expression data sets. Four data sets were profiled on Affymetrix platforms, "Wang" [24], "Loi" [25], "Mainz" [26] and "Frid" [27], while the other two were profiled on Illumina beadarrays, "NCH" [28] and "GH"- a small subset of the data published in [29]. Normalized copy-number calls were available for three data sets: Wang [24], NCH [28] and GH [29]. The "Wang data set" [24] had the largest sample size (209 ER+ samples, 77 ER- samples), and hence was used as the training/discovery set, while the other five data sets were used to evaluate and compare the consistency of activity inference obtained using the different methods.

We also considered five lung cancer/normal expression data sets [30-34]. One data set ("Wachi") consisted of 5 lung cancers and 5 normal samples [30]. Another set ("Su") consisted of 27 matched pairs of normal/cancer lung tissue (54 samples in total) [31]. The third set ("Landi") consisted of 49 normal lung samples and 58 lung cancers [32]. The fourth set ("Su") consisted of 18 lung cancers and 12 normal lung samples [33] and finally the fifth set ("Lu") consisted of 60 matched lung cancer/normal pairs. All of these expression sets used the Affymetrix Human Genome U133A or U133 Plus 2.0 Array. We used the "Landi" set for the training/discovery of the pruned relevance network and the rest as validation studies.

### Mammogram density scoring

Mammograms consisted of original standard mediolateral oblique and craniocaudal views and mammographic density was scored by an independent consultant radiologist. As all patients had been diagnosed with malignancy, the density of the tumour itself was scored on a scale from 1-5 (5 being the most dense and one being the least) without inclusion of normal breast tissue.

### DART: Denoising Algorithm based on Relevance network Topology

We assume a given pathway *P *with prior information consisting of genes which are upregulated in response to pathway activation *P*_*U *_and genes which are downregulated *P*_*D*_. Let *n*_*U *_and *n*_*D *_denote the corresponding number of up and downregulated genes in the pathway. We point out that for the given prior pathway information, *n*_*U *_or *n*_*D *_may be zero, in other words, DART does not require both to be non-zero. Given a gene expression data set *X *of *G *genes and *n*_*S *_samples, unrelated to this prior information, we wish to evaluate a level of pathway activation for each sample in *X*.

Before estimating pathway activity we argue that the prior information needs to be evaluated in the context of the given data. For example, if two genes are commonly upregulated in response to pathway activation and if this pathway is indeed activated in a given sample, then the expectation is that these two genes are also upregulated in this sample relative to samples which do not have this pathway activated. In fact, given the set of a priori upregulated genes *P*_*U *_we would expect that these genes are all correlated across the sample set being studied, provided of course that this (i) prior information is reliable and relevant in the present biological context and (ii) that the pathway shows differential activity across the samples. Thus, we propose the following strategy to arrive at improved estimates of pathway activity:

1. Compute and construct a relevance correlation network of all genes in pathway *P*.

2. Evaluate a consistency score of the prior regulatory information of the pathway by comparing the pattern of observed gene-gene correlations to those expected under the prior.

3. If the consistency score is higher than expected by random chance, the consistent prior information may be used to infer pathway activity. The inconsistent prior information must be removed by pruning the relevance network. This is the denoising step.

4. Estimate pathway activity from computing a metric over the largest connected component of the pruned network.

We consider three different variations of the above algorithm in order to address two theoretical questions: (i) Does evaluating the consistency of prior information in the given biological context matter and does the robustness of downstream statistical inference improve if a denoising strategy is used? (ii) Can downstream statistical inference be improved further by using metrics that recognise the network topology of the underlying pruned relevance network? We therefore consider one algorithm in which pathway activity is estimated over the unpruned network using a simple average metric ("UPR-AV") and two algorithms that estimate activity over the pruned network but which differ in the metric used: in one instance we average the expression values over the nodes in the pruned network ("PR-AV"), while in the other case we use a weighted average ("DART") where the weights reflect the degree of the nodes in the pruned network. The rationale for this is that the more nodes a given gene is correlated with, the more likely it is to be relevant and hence the more weight it should receive in the estimation procedure. This metric is equivalent to a summation over the edges of the relevance network and therefore reflects the underlying topology [5].

Next, we clarify how DART was applied to the various signatures considered in this work. In the case of the perturbation signatures, DART was applied to the combined upregulated and downregulated gene sets, as described above. In the case of the Netpath signatures (which were more numerous) we were interested in also investigating if the algorithms performed differently depending on the gene subset considered (i.e if up or downregulated set). Thus, in the case of the Netpath signatures we applied DART to the up and down regulated gene sets separately. This strategy was also partly motivated by the fact that most of the Netpath signatures had relatively large up and downregulated gene subsets.

### Constructing expression relevance networks

Given the set of transcriptionally regulated genes and a gene expression data set, we compute Pearson correlations between every pair of genes. The Pearson correlation coefficients were then transformed using Fisher's transform

(1) $$y_{ij}=\frac{1}{2}\log\frac{1+c_{ij}}{1-c_{ij}}$$

where *c*_*ij *_is the Pearson correlation coefficient between genes *i *and *j*, and where *y*_*ij *_is, under the null hypothesis, normally distributed with mean zero and standard deviation $1∕\sqrt{n_{s}-3}$ with *n*_*s *_the number of tumour samples. From this, we then derive a corresponding p-value matrix. To estimate the false discovery rate (FDR) we needed to take into account the fact that gene pair correlations do not represent independent tests. Thus, we randomly permuted each gene expression profile across tumour samples (a Monte Carlo run) and selected a p-value threshold (0.0001) that yielded a negligible average FDR (an average of less than 1 false positive as averaged over 1000 Monte Carlo runs). Gene pairs with correlations that passed this p-value threshold were assigned an edge in the resulting relevance expression correlation network.

The estimation of P-values assumes normality under the null, and while we observed marginal deviations from a normal distribution (data not shown), the above FDR estimation procedure is equivalent to one which works on the absolute values of the statistics *y*_*ij*_. This is because the P-values and absolute valued statistics (|*y*_*ij*_|) are related through a monotonic transformation, thus the FDR estimation procedure we used does not require the normality assumption.

### Evaluating significance and consistency of relevance networks

The consistency of the derived relevance network with the prior pathway regulatory information was evaluated as follows: given an edge in the derived network we assigned it a binary weight (1,-1) depending on whether the correlation between the two genes is positive (1) or negative (-1). This binary weight can then be compared with the corresponding weight prediction made from the prior, namely a 1 if the two genes are either both upregulated or both downregulated in response to the oncogenic perturbation, or -1 if they are regulated in opposite directions. Thus, an edge in the network is consistent if the sign is the same as that of the model prediction. A consistency score for the observed network is obtained as the fraction of consistent edges. To evaluate the significance of the consistency score we used a randomisation approach. Specifically, for each edge in the network the binary weight was drawn from a binomial distribution with the binomial probability estimated from the whole data set. We estimated the binomial probability of a positive weight (1) as the fraction of positive pairwise correlations among all significant pairwise correlations. A total of 1000 randomisations were performed to derive a null distribution for the consistency score, and a p-value was computed as the fraction of randomisations with a consistency score higher than the observed one.

### Pathway activation metrics

First, we define the single-gene based pathway activation metric. This metric is similar to the subnetwork expression metric used in the context of protein-interaction networks [9]. The metric over the network (pruned or unpruned) of size *M *is defined as,

(2) $${\vec{s}}_{AV}=\frac{1}{\sqrt{M}}\underset{i\in N}{\sum }\sigma_{i}{\vec{z}}_{i}$$

where ${\vec{z}}_{i}$ denotes the z-score normalised (mean zero and unit variance) expression profile of gene *i *across the samples and *σ*_*i *_denotes the sign of pathway activation (from the prior information), i.e *σ*_*i *_= 1 if upregulated upon activation, *σ*_*i *_= -1 if downregulated. Thus, this metric is a simple average over the genes in the network and does not take the underlying topology into account. An alternative is to weight each gene by the number of its neighbors in the network

(3) $${\vec{s}}_{W\;AV}=\frac{1}{\sqrt{\Sigma_{i\in N}k_{i}^{2}}}\underset{i\in N}{\sum }\sigma_{i}k_{i}{\vec{z}}_{i}$$

where *k*_*i *_is the number of neighbors of gene *i *in the network. Normally, this would include neighbors that are both in *P*_*U *_and in *P*_*D*_. The normalisation factor ensures that *s*_*W AV*_, if interpreted as a random variable, is of unit variance.

### Simulated data

To test the principles on which our algorithm is based we generated synthetic gene expression data as follows. We generated a toy data matrix of dimension 24 genes times 100 samples. We assume 40 samples to have no pathway activity, while the other 60 have variable levels (we assume 3 levels) of pathway activity. The 24 genes are all assumed to be part of a given pathway, but only 3 are assumed to faithfully represent the pathway in the synthetic data set. Specifically, the data is simulated as

$$\begin{array}{c} X_{1s}\sim \delta_{s\leq 40}N\left(0,\sigma_{1}\right)+\delta_{s>40}N\left(2,\sigma_{1}\right)\\ X_{2s}\sim \delta_{\left(s\leq 40\right)\cup \left(60<s\leq 80\right)}N\left(0,\sigma_{1}\right)+\delta_{\left(40<s\leq 60\right)\cup \left(80<s\right)}N\left(2,\sigma_{1}\right)\\ X_{3s}\sim \delta_{s\leq 80}N\left(0,\sigma_{1}\right)+\delta_{80<s}\left(2,\sigma_{1}\right) \end{array}$$

where *N *denotes the normal distribution of the given mean and standard deviation (*σ*_1 _= 0.25), and where *δ *is the Kronecker delta such that *δ*_*x *_*= *1 if and only if condition *x *is true. The rest of the genes are modelled from the same distributions but with *σ*_2_(= 3) replacing *σ*_1_(= 0.25), thus these genes are subject to large variability and don't provide faithful representations of the pathway. Thus, in this synthetic data set all genes are assumed upregulated in a proportion of the samples with pathway activity but only a relatively small number are not subject to other sources of variation. We point out that the more general case of some genes being upregulated and others being downregulated is in fact subsumed by the previous model, since the significance analysis of correlations or anticorrelations is identical and since the pathway activation metric incorporates the directionality explicitly through a change in the sign of the contributing genes.

We also consider an alternative scenario in which only 6 genes are upregulated in the 60 samples. Of the 6 genes, 3 are generated as above with *σ*_1 _= 0.25 and the other 3 with *σ*_2 _= 3. The rest of genes are modelled as *N*(0, 2) and are therefore not discriminatory. We call this synthetic data set "SimSet2", while the previous one we refer to as "SimSet1".

The algorithms described previously are then applied to the simulated data to infer pathway activity levels. To objectively compare the different algorithms we apply a variational Bayesian Gaussian Mixture Model [35] to the pathway activity level. The variational Bayesian approach provides an objective estimate of the number of clusters in the pathway activity level profile. The clusters map to different activity levels and the cluster with the lowest activity level defines the "ground state" of no activation. Hence we can compare the different algorithms in terms of the accuracy of correctly assigning samples with no activity to the ground state and samples with activity to any of the higher levels, which will depend on the predicted pathway activity levels.

### Evaluation based on pathway correlations

One way to evaluate and compare the different estimation procedures is to consider pairs of pathways for which the corresponding estimated activites are significantly correlated in a training set and then see if the same pattern is observed in a series of validation sets. Thus, significant pathway correlations derived from a given discovery/training set ("train") can be viewed as hypotheses, which if true, must validate in the independent data sets. We thus compare the algorithms in their ability to identify pathway correlations which are also valid in independent data.

Specifically, for a given pathway activity estimation algorithm and for a given pair of pathways *(i,j)*, we first correlate the pathway activation levels using a linear regression model. Under the null, the z-scores are distributed according to t-statistics, therefore we let *t*_*ij *_denote the t-statistic and *p*_*ij *_the corresponding *P-*value. We declare a significant association as one with *p*_*ij *_< 0.05, and if so it generates a hypothesis. To test the consistency of the predicted interpathway Pearson correlation in the validation data sets *D*, we use the following performance measure *V*_*ij*_:

(4) $$V_{ij}=\underset{d\in D}{\sum }\sigma_{ij}^{\left(d\right)}\left|t_{ij}^{\left(d\right)}\right|S\left(p_{ij}^{\left(d\right)}\right)$$

where the summation is over the validation sets, *S *is the threshold function of *p*_*ij *_defined by

(5) $$S\left(p_{ij}\right)=\left\{\begin{array}{cc} 1 & \text{if}\;p_{ij}\leq 0.05\\ 0 & \text{if}\;p_{ij}>0.05 \end{array}\right)$$

and where

(6) $$\sigma_{ij}^{\left(d\right)}=\left\{\begin{array}{cc} \;1 & \text{if}\;\text{sign}\left(t_{ij}^{\left(\text{train}\right)}\right)=\text{sign}\left(t_{ij}^{\left(d\right)}\right),d\in D\\ -1 & \text{if}\;\text{sign}\left(t_{ij}^{\left(\text{train}\right)}\right)=-\text{sign}\left(t_{ij}^{\left(d\right)}\right),d\in D \end{array}\right)$$

In the above, $t_{ij}^{\left(d\right)}$ is the t-statistic of interpathway correlation estimated in validation set *d *∈ *D *and $\left|t_{ij}^{\left(d\right)}\right|$ de-notes its absolute value. Thus, the quantity *V*_*ij *_takes into account the significance of the correlation between the pathways (through the threshold function *S)*, penalizes the score if the directionality of correlation is opposite to that predicted (through $\sigma_{ij}^{\left(d\right)}$) and weighs in the magnitude of the correlation association $\left(\left|t_{ij}^{\left(d\right)}\right|\right)$. For each method, we thus obtain a set of hypotheses $H^{\left(m\right)}=\left\{\left(i,j\right):p_{ij}^{\left(\text{train}\right)}<0.05\right\}$ and consistency scores over $H^{\left(m\right)}:V^{\left(m\right)}=\left\{V_{ij}^{\left(m\right)}:\left(i,j\right)\in H^{\left(m\right)}\right\}$. Finally, an objective comparison between two different methods (*m*_1_, *m*_2_) for pathway activity estimation can be achieved by comparing the distribution of $V^{\left(m_{1}\right)}$ to that of $V^{\left(m_{2}\right)}$ over the common hypothesis space i.e $H^{\left(m_{1}\right)}\cap H^{\left(m_{2}\right)}$. For this we used a two-tailed paired Wilcoxon test.

## Results and Discussion

**Figure 1:**
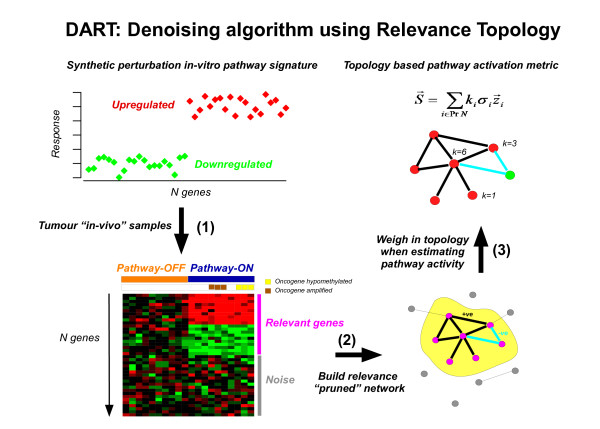
**The DART algorithm**. Flowchart describing the steps in DART (Denoising algorithm based on Relevance network Topology). (1) A synthetic perturbation mRNA signature (e.g derived from an in-vitro cell model, or from a curated list) is evaluated across clinical tumour specimens of a given cancer where pathway activity estimates are sought. Only a subset of genes will show correlations in response to differential pathway activation across tumours. (2) Construction of relevance correlation pruned network as the maximally connected component where all edges reflect correlations that are consistent with the prior information given by the synthetic perturbation signature. (3) Pathway activation over pruned network via a topology based metric, which gives more weight to the hubs in the network. The hypothesis is that correlation hubs represent corresponding pathway activity more faithfully than non-hubs.

We argue that more robust statistical inferences regarding pathway activity levels and which use prior knowledge from pathway databases can be obtained by first evaluating if the prior information is consistent with the data being investigated (Figure 1). If the expression level of a certain set of genes faithfully represents pathway activity and if these genes are commonly upregulated in response to pathway activation, then one would expect these genes to show significant correlations at the level of gene expression across a sample set, provided of course that differential activity of this pathway accounts for a proportion of the data variance. Thus, one may use a gene expression data set to evaluate the consistency of the prior information and to filter out the information which represents noise.

### Simulated Data

**Figure 2:**
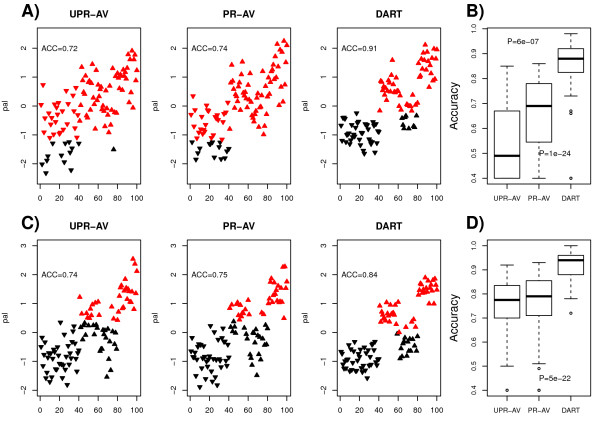
**Simulated data**. **A) & C) **Predicted pathway activity levels (pal) (y-axis) against sample-ID (x-axis) for specific runs of the simulated data. Pathway activity levels were estimated separately using the three algorithms (i) unpruned average metric (UPR-AV), (ii) pruned average metric (PR-AV) and (iii) pruned weighted average metric (DART). The variational Bayesian clustering approach was used to infer the clusters in these pathway activity level profiles. Black denotes the inferred cluster with lowest mean pathway activity level, red denotes samples assigned to higher level clusters. Downward pointing triangles denote the samples with no true pathway activity (samples 1-40), upward pointing triangles denote the samples with true pathway activity (albeit variable levels) (samples 41-100). The proportion of correctly assigned samples is given (Accuracy). A) refers to SimSet2 and C) refers to SimSet1. **B) & D) **Comparison of classification accuracies (y-axis) of the three algorithms (x-axis) over 100 simulations. B) SimSet2. Wilcoxon-test P-values given are between UPR-AV and PR-AV, and between PR-AV and DART. C) SimSet1. Wilcoxon-test P-value is between PR-AV and DART.

To test the principle (Figure 1) we first generated synthetic data where we know which samples have a hypothetical pathway activated and others where the pathway is switched off (Methods). We considered two different simulation scenarios as described in Methods to represent two different levels of noise in the data. Next, we applied three different methods to infer pathway activity, one which simply averages the expression profiles of each gene in the pathway (UPR-AV), one which infers a correlation relevance network, prunes the network to remove inconsistent prior information and estimates activity by averaging the expression values of the genes in the maximally connected component of the pruned network (PR-AV). The third method also generates a pruned network and estimates activity over the maximally connected subnetwork but does so by a weighted average where the weights are directly given by the degrees of the nodes (DART). To objectively compare the different algorithms, we applied a variational Bayesian clustering algorithm [35,36] to the one-dimensional estimated activity profiles to identify the different levels of pathway activity. The variational Bayesian approach was used over the Bayesian Information Criterion or the Akaike Information Criterion, since it is more accurate for model selection problems, particularly in relation to estimating the number of clusters [35,36]. We then assessed how well samples with and without pathway activity were assigned to the respective clusters, with the cluster of lowest mean activity representing the ground state of no pathway activity. Examples of specific simulations and inferred clusters in the two different noisy scenarios are shown in Figures 2A &2C. We observed that in these specific examples, DART assigned samples to their correct pathway activity level much more accurately than either UPR-AV or PR-AV, owing to a much cleaner estimated activation profile. Average performance over 100 simulations confirmed the much higher accuracy of DART over both PR-AV and UPR-AV (Figures 2B &2D). Interestingly, while PR-AV performed significantly better than UPR-AV in simulation scenario 2 (SimSet2), it did not show appreciable improvement in SimSet1 (Figures 2B &2D). The key difference between the two scenarios is in the number of genes that are assumed to represent pathway activity with all genes assumed relevant in SimSet1, but only a few being relevant in SimSet2. Thus, the improved performance of PR-AV over UPR-AV in SimSet2 is due to the pruning step which removes the genes that are not relevant in SimSet2.

### Improved prediction of natural pathway perturbations

Given the improved performance of DART over the other two methods in the synthetic data, we next explored if this also held true for real data. We thus collected perturbation signatures of three well-known cancer genes and which were all derived from cell-line models. Specifically, the genes and cell-lines were *ERBB2 *(ER+ breast cancer cell line), *MYC *(human mammary epithelial cells) and *TP53 *(lung cell line) [1,17,22]. We applied each of the three algorithms to these perturbation signatures in the largest of the breast cancer sets (Wang data set in the case of *ERBB2, MYC*) and also one of the largest lung cancer sets (Landi set in the case of *TP53*) to learn the corresponding unpruned and pruned networks. Using these networks we then estimated pathway activity in the same sets as well as in the independent validation sets. We evaluated the three algorithms in their ability to correctly predict pathway activation status in clinical tumour specimens. In the case of *ERBB2*, amplification of the *ERBB2 *locus occurs in only a subset of breast cancers (HER2+ subtype), which have a characteristic transcriptomic signature [37]. Specifically, we would expect HER2+ breast cancers defined by the intrinsic subtype transcriptomic classification to have higher *ERBB2 *pathway activity than basal breast cancers which are HER2- [37]. Thus, pathway activity estimation algorithms which predict larger differences between HER2+ and basal breast cancers indicate improved pathway activity inference. Similarly, we would expect breast cancer samples with amplification of *MYC *(a common genomic abnormality in breast cancer) to exhibit higher levels of *MYC*-specific pathway activity. Finally, *TP53 *inactivation, either through mutation or genomic loss, is a common genomic abnormality present in most cancers. Thus, *TP53 *activation levels should be significantly lower in lung cancers compared to respective normal tissue.

**Figure 3:**
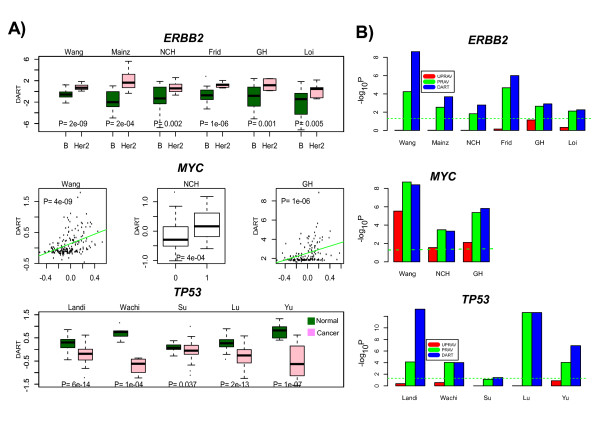
**Predicting pathway activity in tumours**. **A) **Upper panel: predicted DART *ERBB2 *pathway activation scores in the basal (B) and HER2+ subtypes of ER-breast cancer and across six different breast cancer cohorts. P-values are from a one-tailed t-test since activity is predicted to be higher in the HER2+ subtype. The pruned network was only learned *once*, from the Wang set, and this same network was then used to compute pathway activity in the other 5 cohorts (Mainz,NCH,Frid,GH,Loi). Middle panel: predicted DART *MYC *activity scores across three breast cancer cohorts with combined expression and copy-number information (Wang, NCH, Loi). DART scores are plotted against segmented *MYC *copy number value (Wang,GH) or called copy-number state (NCH,1 = gain,0 = no-change or loss). One tailed P-value was estimated empirically from linear regression on permuted sample labels (Wang,GH) or from a one-tailed t-test (NCH). As in A), pruned network was learned in Wang, and same network used in NCH and GH. Lower panel: predicted DART *TP53 *activity scores in three lung cancer/normal data sets (Landi,Wachi,Su). Pruned network was learned in Landi, and this network tested in Wachi and Su. P-values are from a one-tailed t-test. We point out that DART learns the pruned network without using phenotypic sample information (i.e Basal/Her2, Copy-number, Normal/Cancer status), thus the results in Wang and Landi sets are not due to selection. **B) **For each of *ERBB2, MYC *and *TP53 *and for each data set we compare the significance of the associations (-log_10_(*P - value*)) between the three methods (UPR-AV,PR-AV,DART). The green dashed line represents the line where *P *= 0.05 and values above it are declared significant.

Of the 14 data sets analysed, encompassing three different perturbation signatures, DART predicted with statistical significance the correct association in all 14 (*P *< 0.05 in all 14 cases) (Figure 3A). Specifically, *ERBB2 *pathway activity was significantly higher in ER-/HER2+ breast cancer compared to the ER-/basal subtype, *MYC *activity was significantly higher in breast tumours with *MYC *copy number gain, and *TP53 *activity was significantly less in lung cancers (frequent *TP53 *inactivation) compared to normal lung tissue. In contrast, using the other two methods (PR-AV, UPR-AV) predictions were either less significant (PR-AV) or less robust (UPR-AV): we observed many instances where UPR-AV failed to capture the known biological association (Figure 3B).

### Evaluation of Netpath in breast cancer gene expression data

Next, we wanted to evaluate the Netpath resource in the context of breast cancer gene expression data. To this end we applied our algorithm to ask if the genes hypothesized to be up and downregulated in response to pathway stimuli showed corresponding correlations across primary breast cancers, which may therefore indicate potential relevance of this pathway in explaining some of the variation in the data. Because of the large differences in expression between ER+ and ER- breast cancer the evaluation was done for each subtype separately (Table 1). The inferred relevance correlation networks were sparse, specially in ER-breast cancer, and for many pathways a large fraction of the correlations were inconsistent with the prior information. Given the relatively large number of edges in the network even small consistency scores were statistically significant. The analysis did reveal that for some pathways (e.g Notch pathway, BCellReceptor) the prior information was not at all consistent with the expression patterns observed indicating that this specific prior information would not be useful in this context. The specific pruned networks and the genes ranked according to their degree/hubness in the these networks are given in Additional Files 1,2,3,4.

**Table 1:** Netpath consistency scores in breast cancer

		ER-				ER+			
**Pathway**	**nG**	**nE**	**fE**	**fconsE**	**Pval**	**nE**	**fE**	**fconsE**	**Pval**

a6b4	27	33	0.09	0.64	0.05	94	0.27	0.53	0.22
AR	511	6164	0.05	0.55	< 0.001	22486	0.17	0.54	< 0.001
BCellReceptor	396	5324	0.07	0.51	0.10	16503	0.21	0.50	0.17
EGFR1	236	2256	0.08	0.56	< 0.001	5896	0.21	0.54	< 0.001
IL1	231	1926	0.07	0.60	< 0.001	5458	0.21	0.56	< 0.001
IL2	722	18836	0.07	0.54	< 0.001	52916	0.20	0.52	< 0.001
IL3	49	99	0.08	0.64	< 0.001	257	0.22	0.57	0.01
IL4	292	3463	0.08	0.54	< 0.001	9531	0.22	0.53	< 0.001
IL5	167	1109	0.08	0.75	< 0.001	3330	0.24	0.64	< 0.001
IL6	104	250	0.05	0.62	< 0.001	1037	0.19	0.62	< 0.001
IL7	62	189	0.10	0.63	< 0.001	353	0.19	0.59	< 0.001
IL9	24	12	0.04	0.92	< 0.001	47	0.17	0.83	< 0.001
KitReceptor	70	115	0.05	0.79	< 0.001	477	0.20	0.60	< 0.001
Notch	92	313	0.07	0.53	0.13	876	0.21	0.51	0.24
RANKL	69	147	0.06	0.62	< 0.001	394	0.17	0.53	0.14
TCellReceptor	561	11587	0.07	0.59	< 0.001	31820	0.20	0.55	< 0.001
TGFBReceptor	993	21396	0.04	0.53	< 0.001	92352	0.19	0.51	< 0.001
TNFA	801	11226	0.04	0.60	< 0.001	54534	0.17	0.53	< 0.001

For both the ER- and ER+ breast cancer data set [24] and for a number of important Netpath cancer signalling and immune signalling pathways (see Methods), we list some of the network properties of the inferred relevance expression correlation networks. For each molecular pathway we give the number of genes of the pathway present in the expression matrix (nG), the number and fraction of edges (i.e significant pairwise correlations between genes) (nE & fE), the fraction of edges that are consistent with the prior information (fconsE) and the corresponding p-value of significance (Pval). P-values were estimated using 1000 permutations.

### Denoising prior information improves the robustness of statistical inference

**Figure 4:**
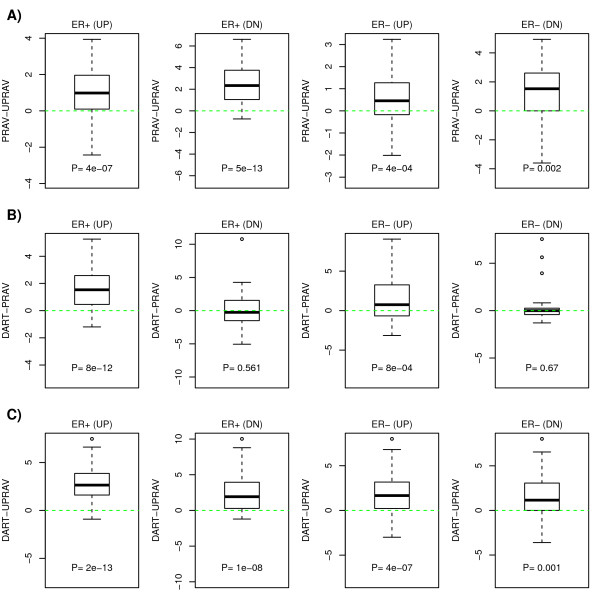
**Consistency of inferred pathway activity correlations**. For each breast cancer subtype (ER+,ER-) and for the up and down transcriptional modules we compare the consistency scores of predicted Netpath pathway correlations evaluated over the 5 validation sets for the three different pathway activity estimation algorithms (UPR-AV,PR-AV,DART). The up and downregulated transcriptional modules for each pathway were inferred from Wang data set and the significant inter-pathway correlations constituted our hypotheses to be tested in the 5 validation sets. Consistency scores reflect significance as well as consistency of directionality and magnitude of correlations in the validation sets. **A) **Boxplot of the difference in consistency scores between PR-AV and UPR-AV methods (PR-AV minus UPR-AV). **B) **Boxplot of the difference in consistency scores between DART and PR-AV methods (DART minus PR-AV). **C) **Boxplot of the difference in consistency scores between DART and UPR-AV methods (DART minus UPR-AV). P-values are from a two-tailed paired Wilcoxon rank sum test.

Another strategy to evaluate and compare the different algorithms is in their ability to make correct predictions about pathway correlations. Knowing which pathways correlate or anticorrelate in a given phenotype can provide important biological insights [5]. Thus, having estimated the pathway activity levels in our training breast cancer set we next identified the statistically significant correlations (or anticorrelations) between pathways in this same set. We treat these significant correlations as hypotheses. For each significant pathway pair we then computed a consistency score over the 5 validation sets (Methods) and compared these consistency scores between the three different algorithms. The consistency scores reflect the overall significance, directionality and magnitude of the predicted correlations in the validation sets (Figure 4). We found that DART significantly improved the consistency scores over the method that did not implement the denoising step (UPR-AV), for both breast cancer subtypes as well as for the up and down regulated transcriptional modules (Figure 4A).

### Expression correlation hubs improve pathway activity estimates

Using the weighted average metric (DART) also improved consistency scores over using an unweighted average (PR-AV), but this was true only for the up regulated modules (Figure 4B). Generally, consistency scores were also higher for the predicted up-regulated modules, which is not surprising given that the Netpath transcriptional modules mostly reflect the effects of positive pathway stimuli as opposed to pathway inhibition. Thus, the better consistency scores for DART over PR-AV indicates that the identified transcriptional hubs in these up-regulated modules (which give more weight to the activity estimates) are of biological relevance. Down regulated genes might reflect further downstream consequences of pathway activity and therefore "hubness" in these modules may be less relevant. Importantly, weighing in hubness in pathway activity estimation also led to stronger associations between predicted *ERBB2 *activity and *ERBB2 *intrinsic subtype (Figure 3B).

### DART compares favourably to supervised methods

**Figure 5:**
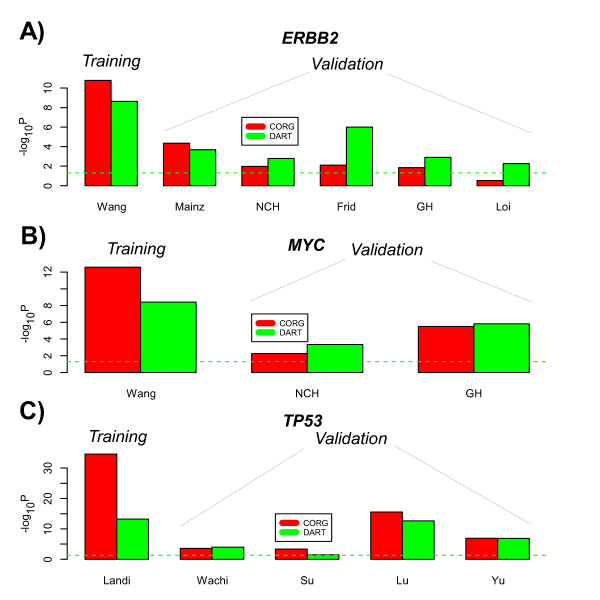
**Comparison of DART with CORG**. Comparison of (unsupervied) DART with (supervised) CORG for the three different perturbation signatures: **A) ***ERBB2 *in ER- breast cancer, **B) ***MYC *in ER+ breast cancer, and **C) ***TP53 *in lung cancer/normal tissue. In the case of *ERBB2 *and *MYC*, Wang was used as the discovery set to learn the associated pruned relevance networks, while for *TP53*, the Landi set was used. The other cohorts are the test sets. y-axis labels the -log10(P-value), reflecting the degree of difference in predicted pathway activity levels between samples with and without the perturbation.

Next, we decided to compare DART to a state of the art algorithm used for pathway activity estimation. Most of the existing algorithms are supervised, such as for example the Signalling Pathway Impact Analysis (SPIA) [12] and the Condition Responsive Genes (CORG) [11] algorithms. SPIA uses the phenotype information from the outset, computing statistics of differential expression for each of the pathway genes between the two phenotypes, and finally evaluates the consistency of these statistics with the topology of the pathway to arrive at an impact score, which informs on differential activity of the pathway between the two phenotypes. However, SPIA is not aimed at identifying a pathway gene subset that could be used to estimate pathway activity at the level of an individual sample, thus precluding a direct comparison with DART. CORG on the other hand, while also being supervised, infers a relevant gene subset, and therefore, like DART, allows pathway activity levels in independent samples to be estimated. Specifically, a comparison can be made between DART and CORG by applying each to the same training set and then evaluating their performance in the independent data sets. We followed this strategy in the context of the *ERBB2, MYC *and *TP53 *perturbation signatures (Figure 5). As expected, owing to its supervised nature, CORG performed better in the three training sets. However, in the 11 independent validation sets (only DART validated successfully *P *< 0.05 in all 11), DART yielded better discriminatory statistics in 7 of these 11 sets (Figure 5). Thus, despite DART being unsupervised in the training set, it achieved comparable performance to CORG in the validation sets.

### DART predicts an association between differential ESR1 signalling and mammographic density

Mammographic density is a well-known risk factor for breast cancer. Indeed, women with high mammo-graphic density (MMD) have an approximately 6-fold higher risk of developing the disease [38]. However, no biological correlates of MMD are known [19]. Therefore there has been a lot of recent interest in obtaining molecular correlates (mRNA expression, SNPs) of mammographic density [19-21]. Based on these studies there is now considerable evidence that dysregulated oestrogen metabolism and signalling may be associated with mammographic density [20], and indeed there have been reports of *ESR1 *expression levels being reduced in breast tissue of high MMD [19].

**Figure 6:**
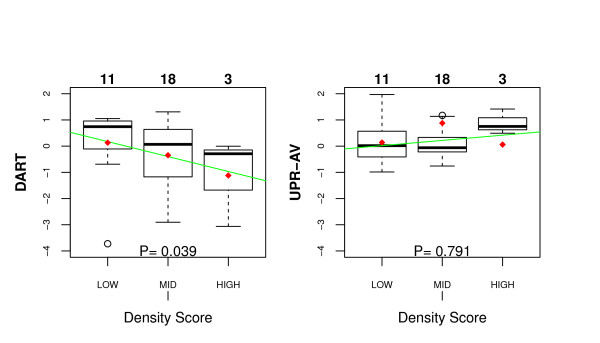
**DART predicts association between ESR1 signalling and mammographic density in ER+ breast cancer**. Predicted DART (left panel) and UPR-AV (right panel) scores across 32 ER+ breast cancers from the GH cohort, for which matched mammographic density scores were available. Density scores were categorized as low, middle or high density. Green line denotes the best least squares fit and red points denote the mean scores in each class. P-values were estimated from permutation of sample labels.

We thus decided to test DART in its ability to detect an entirely novel biological association, specifically we asked if DART could predict an inverse correlation between *ESR1 *signalling activity and MMD. To address this we used the *ESR1 *signature derived in [39]. We verified that this signature was able to discriminate ER+ from ER- tumours in all breast cancer cohorts (Additional File 5), thus confirming that this signature is activated upon *ESR1 *signalling. Next, we applied DART to this signature in the Wang ER+ cohort to learn an associated relevance network for pathway activity estimation. We then estimated pathway actity using this relevance network in the GH ER+ cohort (32 samples), for which MMD scores were available. Of note, DART predicted an inverse correlation between *ESR1 *signalling and MMD (Figure 6). In contrast, not using the denoising step (UPR-AV) failed to pick out this association (Figure 6).

### Discussion

The ability to reliably predict pathway activity of oncogenic and cancer signalling pathways in individual tumour samples is an important goal in cancer genomics. Given that any single tumour is characterised by a large number of genomic and epigenomic aberrations, the ability to predict pathway activity may allow for a more principled approach of identifying driver aberrations as those whose transcriptional fingerprint is present in the mRNA profile of the given tumour. This is critical for assigning patients the appropriate treatments that specifically target those molecular pathways which are functionally disrupted in the patient's tumour. Another important future area of application is in the identification of molecular pathway correlates of cancer imaging traits. Imaging traits, such as mammographic density, may provide important additional information, which is complementary to molecular profiles, but which combined with molecular data may provide critical and novel biological insights.

A large number of algorithms for predicting pathway activity exist and most use prior pathway models obtained through highly curated databases or through *in-vitro *perturbation experiments. A common feature of these methods is the direct application of this prior information in the molecular profiles of the study in question. While this direct approach has been successful in many instances [1], we have also found many examples where it fails to uncover known biological associations (Figure 3). For example, a synthetic perturbation signature of *ERBB2 *activation may not predict the naturally occuring *ERBB2 *perturbation (i.e amplification of the *ERBB2 *locus which in effect defines the *ERBB2 *intrinsic subtype) in primary breast cancers (Figure 3). Similarly, a synthetic perturbation signature for *TP53 *activation was not significantly lower in lung cancer compared to normal lung tissue, despite the fact that *TP53 *inactivation is a frequent event in lung cancer (Figure 3). We argue that this problem is caused by the implicit assumption that all prior information associated with a given pathway is of equal importance or relevance in the biological context of the given study, a context which may be quite different to the biological context in which the prior information was obtained.

To overcome this problem, we propose that the prior information ought to be tested first for its consistency in the data set under study and that pathway activity should be estimated a posteriori using only the prior information that is consistent with the actual data. We point out that this denoising/learning step does not make use of any phenotypic information regarding the samples, and therefore is totally unsupervised. Thus, our approach can be described as unsupervised Bayesian, and Bayesian algorithms using explicit posterior probability models could be implemented. Here, we used a relevance network topology approach to perform the denoising, as implemented in the DART algorithm. Using multiple different *in-vitro *derived perturbation signatures as well as curated transcriptional modules from the Netpath resource on real mRNA expression data, we have shown that DART clearly outperforms a popular model which does not denoise the prior information (Figures 3 &4). Moreover, we have observed that expression correlation hubs, which are inferred as part of DART, improve the consistency scores of pathway activity estimates. This indicates that hubs in relevance networks not only represent more robust markers of pathway activity but that they may also be more important mediators of the functional effects of upstream pathway activity.

It is important to point out again that DART is an unsupervised method for inferring a subset of pathway genes that represent pathway activity. Identification of this gene pathway subset allows estimation of path-way activity at the level of individual samples. Therefore, a direct comparison with the Signalling Pathway Impact Analysis (SPIA) method [12] is difficult, because SPIA does not infer a relevant pathway gene subset, hence not allowing for individual sample activity estimates to be obtained. Thus, instead of SPIA, we compared DART to a different supervised method (CORG) which does infer a pathway gene subset, and which therefore allows single sample pathway activity estimates to be obtained. This comparison showed that in independent data sets, DART performed similarly to CORG (Figure 5). Thus, supervised approaches may not outperform an unsupervised method (here DART) when testing in completely independent data. We also observed that CORG generally yielded very small gene subsets (just a couple of genes) compared to the larger gene subnetworks inferred using DART (Additional File 6). While a small discriminatory gene set may be advantageous from an experimental cost viewpoint, biological interpretation is less clear. For instance, in the case of the *ERBB2, MYC *and *TP53 *perturbation signatures, Gene Set Enrichment Analysis (GSEA) could not be applied to the CORG gene modules since these consisted of too few genes. In contrast, GSEA on the relevance gene subnetworks inferred with DART yielded the expected associations (Additional Files 7,8,9) but also elucidated some novel and biologically interesting associations, such as the association of a tosedostat drug signature with the *MYC *DART module (Additional File 8). A second important difference between CORG and DART is that CORG only ranks genes according to their univariate statistics, while DART ranks genes according to their degree in the relevance subnetwork. Given the importance of hubs in these expression networks, DART thus provides an improved framework for biological interpretation. For instance, the protein kinase *MELK *was the top ranked hub in the *ERBB2 *DART module, suggesting an important role for this downstream kinase in linking cell-growth to the upstream *ERBB2 *perturbation. Interestingly, overexpression of *MELK *is a robust poor prognostic factor in breast cancer [40,41] and may thus contribute to the poor prognosis of HER2+ breast cancers.

Finally, we tested DART in a novel application to multidimensional cancer genomic data, in this instance between matched mRNA expression and imaging traits of clinical breast tumours. Interestingly, DART predicted an inverse correlation between *ESR1 *signalling and MMD in ER+ breast cancer (Figure 6). This association and its directionality is consistent with a study strongly implicating oestrogen metabolism [20] and another reporting an inverse correlation of *ESR1 *expression with MMD [19]. Importantly, not using the denoising step in DART, completely failed to capture this potentially important and biologically plausible association.

In summary, we have shown that the denoising step implemented in DART is critical for obtaining more reliable estimates of molecular pathway activity. It could be argued that a practical drawback of the procedure is the reliance on a relatively large data set (in this context a genome-wide gene expression panel of primary tumours) in order to denoise the prior pathway knowledge. However, large panels of genome-wide molecular data, including expression data of specific cancers, are being generated as part of large international consortia (see e.g [29,42]), and since these large studies use cohorts representative of the disease demographics in question, they constitute ideal data sets to use in the context of DART. Thus, we propose a strategy whereby DART is used to integrate existing pathway databases with these large expression data sets in order to obtain more reliable molecular pathway activity predictions in tumour samples derived from newly diagnosed patients.

## Conclusions

The DART algorithm and strategy advocated here substantially improves unsupervised predictions of pathway activity that are based on a prior model which was learned from a different biological system or context. It will be fruitful to apply DART and further extensions of it in the context of multidimensional cancer ge-nomic data, where reliable and robust molecular pathway correlates of (epi)genomic abnormalities, clinical and imaging traits are urgently needed.

## Abbreviations

ER+: (ER positive breast cancer); ER-: (ER negative breast cancer)

## Competing interests

The authors declare that they have no competing interests.

## Authors' contributions

YJ performed most of the statistical analyses and contributed to the writing of the manuscript. AET conceived the study, performed some of the analyses and wrote the manuscript. KL and AG helped with bioinformatics and data processing. GP and AJ obtained, analysed and scored the mammogram data. AP is the principal investigator at KCL for the Molecular Taxonomy of Breast Cancer International Consortium programme (METABRIC, Cancer Research UK), which funded the gene array analysis. TN designed the original concept of correlating and integrating imaging with genomic analyses, obtained funding and contributed to final writing of manuscript. All authors read and approved the final manuscript.

## Supplementary Material

Additional file 1 **DART modules for Netpath pathways in ER+ breast cancer**. Tables listing the pruned, consistent, maximally connected networks inferred from the application of DART to the transcriptional up and downregulated modules from Netpath in the ER+ subset of the Wang data set. Click here for file

Additional file 2 **DART modules for Netpath pathways in ER- breast cancer**. Tables listing the pruned, consistent, maximally connected networks inferred from the application of DART to the transcriptional up and downregulated modules from Netpath in the ER- subset of the Wang data set. Click here for file

Additional file 3 **Hub genes in DART modules in ER+ breast cancer**. Genes in pruned Netpath pathway networks ranked according to their degree in the network. Click here for file

Additional file 4 **Hub genes in DART modules in ER- breast cancer**. Genes in pruned Netpath pathway networks ranked according to their degree in the network. Click here for file

Additional file 5 **DART ESR1 module in breast cancer**. Boxplots comparing predicted pathway activities of the Doane *ESR1 *signature in ER+ versus ER- tumours in the six different breast cancer cohorts. P-values from a t-test are given. Click here for file

Additional file 6 **Comparison of module genes between CORG and DART**. Table comparing the number of genes in the CORG and DART predictors of pathway activity. Click here for file

Additional file 7 **GSEA table for DART ERBB2 module genes**. Gene Set Enrichment Analysis table for the *ERBB2 *DART gene modules (relevance subnetworks). P-values are from a one-tailed hypergeometric test using the genes on the Affy array (Wang data set) as the null background. Click here for file

Additional file 8 **GSEA table for DART MYC module genes**. Gene Set Enrichment Analysis table for the *MYC *DART gene modules (relevance subnetworks). P-values are from a one-tailed hypergeometric test using the genes on the Affy array (Wang data set) as the null background. Click here for file

Additional file 9 **GSEA table for DART TP53 module genes**. Gene Set Enrichment Analysis table for the *MYC *DART gene modules (relevance subnetworks). P-values are from a one-tailed hypergeometric test using the genes on the Affy array (Wang data set) as the null background. Click here for file

## References

[B1] Bild AH Yao G Chang JT Wang Q Potti A Oncogenic pathway signatures in human cancers as a guide to targeted therapies Nature 2006 439 353 357 10.1038/nature04296 16273092

[B2] Ein-Dor L Kela I Getz G Givol D Domany E Outcome signature genes in breast cancer: is there a unique set? Bioinformatics 2005 21 171 178 10.1093/bioinformatics/bth469 15308542

[B3] Chang JT Carvalho C Mori S Bild AH Gatza ML A genomic strategy to elucidate modules of oncogenic pathway signaling networks Mol Cell 2009 34 104 114 10.1016/j.molcel.2009.02.030 19362539 PMC2694616

[B4] Segal E Sirlin CB Ooi C Adler AS Gollub J Decoding global gene expression programs in liver cancer by noninvasive imaging Nat Biotechnol 2007 25 675 680 10.1038/nbt1306 17515910

[B5] Teschendorff AE Gomez S Arenas A El-Ashry D Schmidt M Improved prognostic classification of breast cancer defined by antagonistic activation patterns of immune response pathway modules BMC Cancer 2010 10 604 10.1186/1471-2407-10-604 21050467 PMC2991308

[B6] Subramanian A Tamayo P Mootha VK Mukherjee S Ebert BL Gene set enrichment analysis: a knowledge-based approach for interpreting genome-wide expression profiles Proc Natl Acad Sci USA 2005 102 15545 15550 10.1073/pnas.0506580102 16199517 PMC1239896

[B7] Tian L Greenberg SA Kong SW Altschuler J Kohane IS Discovering statistically significant pathways in expression profiling studies Proc Natl Acad Sci USA 2005 102 13544 13549 10.1073/pnas.0506577102 16174746 PMC1200092

[B8] Draghici S Khatri P Tarca AL Amin K Done A A systems biology approach for pathway level analysis Genome Res 2007 17 1537 1545 10.1101/gr.6202607 17785539 PMC1987343

[B9] Chuang HY Lee E Liu YT Lee D Ideker T Network-based classification of breast cancer metastasis Mol Syst Biol 2007 3 140 17940530 10.1038/msb4100180 PMC2063581

[B10] Ulitsky I Shamir R Identification of functional modules using network topology and high-throughput data BMC Syst Biol 2007 1 8 10.1186/1752-0509-1-8 17408515 PMC1839897

[B11] Lee E Chuang HY Kim JW Ideker T Lee D Inferring pathway activity toward precise disease classification PLoS Comput Biol 2008 4 e1000217 10.1371/journal.pcbi.1000217 18989396 PMC2563693

[B12] Tarca AL Draghici S Khatri P Hassan SS Mittal P A novel signaling pathway impact analysis Bioinformatics 2009 25 75 82 10.1093/bioinformatics/btn577 18990722 PMC2732297

[B13] Heiser LM Wang NJ Talcott CL Laderoute KR Knapp M Integrated analysis of breast cancer cell lines reveals unique signaling pathways Genome Biol 2009 10 R31 10.1186/gb-2009-10-3-r31 19317917 PMC2691002

[B14] Komurov K White MA Ram PT Use of data-biased random walks on graphs for the retrieval of context-specific networks from genomic data PLoS Comput Biol 2010 6 10.1371/journal.pcbi.1000889 PMC2924243 20808879

[B15] Kandasamy K Mohan SS Raju R Keerthikumar S Kumar GS Netpath: a public resource of curated signal transduction pathways Genome Biol 2010 11 R3 10.1186/gb-2010-11-1-r3 20067622 PMC2847715

[B16] Hoadley KA Weigman VJ Fan C Sawyer LR He X Egfr associated expression profiles vary with breast tumor subtype BMC Genomics 2007 8 258 10.1186/1471-2164-8-258 17663798 PMC2014778

[B17] Creighton CJ Hilger AM Murthy S Rae JM Chinnaiyan AM Activation of mitogen-activated protein kinase in estrogen receptor alpha-positive breast cancer cells in vitro induces an in vivo molecular phenotype of estrogen receptor alpha-negative human breast tumors Cancer Res 2006 66 3903 3911 10.1158/0008-5472.CAN-05-4363 16585219

[B18] Majumder PK Febbo PG Bikoff R Berger R Xue Q mtor inhibition reverses akt-dependent prostate intraepithelial neoplasia through regulation of apoptotic and hif-1-dependent pathways Nat Med 2004 10 594 601 10.1038/nm1052 15156201

[B19] Haakensen VD Biong M Lingjærde OC Holmen MM Frantzen JO Expression levels of uridine 5'-diphospho-glucuronosyltransferase genes in breast tissue from healthy women are associated with mammographic density Breast Cancer Res 2010 12 R65 10.1186/bcr2632 20799965 PMC2949660

[B20] Li J Eriksson L Humphreys K Czene K Liu J Genetic variation in the estrogen metabolic pathway and mammographic density as an intermediate phenotype of breast cancer Breast Cancer Res 2010 12 R19 10.1186/bcr2488 20214802 PMC2879563

[B21] Lindstroem S Vachon CM Li J Varghese J Thompson D Common variants in znf365 are associated with both mammographic density and breast cancer risk Nat Genet 2011 43 185 187 10.1038/ng.760 21278746 PMC3076615

[B22] Kannan K Amariglio N Rechavi G Jakob-Hirsch J Kela I Dna microarrays identification of primary and secondary target genes regulated by p53 Oncogene 2001 20 2225 2234 10.1038/sj.onc.1204319 11402317

[B23] Vogelstein B Kinzler KW Cancer genes and the pathways they control Nat Med 2004 10 789 799 10.1038/nm1087 15286780

[B24] Wang Y Klijn JG Zhang Y Sieuwerts AM Look MP Gene-expression profiles to predict distant metastasis of lymph-node-negative primary breast cancer Lancet 2005 365 671 679 15721472 10.1016/S0140-6736(05)17947-1

[B25] Loi S Haibe-Kains B Desmedt C Lallemand F Tutt AM Definition of clinically distinct molecular subtypes in estrogen receptor-positive breast carcinomas through genomic grade J Clin Oncol 2007 25 1239 1246 10.1200/JCO.2006.07.1522 17401012

[B26] Schmidt M Böhm D von Törne C Steiner E Puhl A The humoral immune system has a key prognostic impact in node-negative breast cancer Cancer Res 2008 68 5405 5413 10.1158/0008-5472.CAN-07-5206 18593943

[B27] Chin K DeVries S Fridlyand J Spellman PT Roydasgupta R Genomic and transcriptional aberrations linked to breast cancer pathophysiologies Cancer Cell 2006 10 529 541 10.1016/j.ccr.2006.10.009 17157792

[B28] Blenkiron C Goldstein LD Thorne NP Spiteri I Chin SF Microrna expression profiling of human breast cancer identifies new markers of tumor subtype Genome Biol 2007 8 R214 10.1186/gb-2007-8-10-r214 17922911 PMC2246288

[B29] Holland DG Burleigh A Git A Goldgraben MA Perez-Mancera PA Znf703 is a common luminal b breast cancer oncogene that differentially regulates luminal and basal progenitors in human mammary epithelium EMBO Mol Med 2011 3 167 180 10.1002/emmm.201100122 21337521 PMC3395113

[B30] Wachi S Yoneda K Wu R Interactome-transcriptome analysis reveals the high centrality of genes differentially expressed in lung cancer tissues Bioinformatics 2005 21 4205 4208 10.1093/bioinformatics/bti688 16188928 PMC4631381

[B31] Su LJ Chang CW Wu YC Chen KC Lin CJ Selection of ddx5 as a novel internal control for q-rt-pcr from microarray data using a block bootstrap re-sampling scheme BMC Genomics 2007 8 140 10.1186/1471-2164-8-140 17540040 PMC1894975

[B32] Landi MT Dracheva T Rotunno M Figueroa JD Liu H Gene expression signature of cigarette smoking and its role in lung adenocarcinoma development and survival PLoS One 2008 3 e1651 10.1371/journal.pone.0001651 18297132 PMC2249927

[B33] Yu K Ganesan K Tan LK Laban M Wu J A precisely regulated gene expression cassette potently modulates metastasis and survival in multiple solid cancers PLoS Genet 2008 4 e1000129 10.1371/journal.pgen.1000129 18636107 PMC2444049

[B34] Lu TP Tsai MH Lee JM Hsu CP Chen PC Identification of a novel biomarker, sema5a, for non-small cell lung carcinoma in nonsmoking women Cancer Epidemiol Biomarkers Prev 2010 19 2590 2597 10.1158/1055-9965.EPI-10-0332 20802022

[B35] Teschendorff AE Wang Y Barbosa-Morais NL Brenton JD Caldas C A variational bayesian mixture modelling framework for cluster analysis of gene-expression data Bioinformatics 2005 21 3025 3033 10.1093/bioinformatics/bti466 15860564

[B36] Attias H Inferring parameters and structure of latent variable models by variational bayes Proceedings of the 15th Conference on Uncertainty in Artificial Intelligence 1999

[B37] Hu Z Fan C Oh DS Marron JS He X The molecular portraits of breast tumors are conserved across microarray platforms BMC Genomics 2006 7 96 10.1186/1471-2164-7-96 16643655 PMC1468408

[B38] Stone J Ding J Warren RM Duffy SW Hopper JL Using mammographic density to predict breast cancer risk: dense area or percentage dense area Breast Cancer Res 2010 12 R97 10.1186/bcr2778 21087468 PMC3046440

[B39] Doane AS Danso M Lal P Donaton M Zhang L An estrogen receptor-negative breast cancer subset characterized by a hormonally regulated transcriptional program and response to androgen Oncogene 2006 25 3994 4008 10.1038/sj.onc.1209415 16491124

[B40] Teschendorff AE Naderi A Barbosa-Morais NL Caldas C Pack: Profile analysis using clustering and kurtosis to find molecular classifiers in cancer Bioinformatics 2006 22 2269 2275 10.1093/bioinformatics/btl174 16682424

[B41] Pickard MR Green AR Ellis IO Caldas C Hedge VL Dysregulated expression of fau and melk is associated with poor prognosis in breast cancer Breast Cancer Res 2009 11 R60 10.1186/bcr2350 19671159 PMC2750122

[B42] Research TCGA Integrated genomic analyses of ovarian carcinoma Nature 2011 474 609 615 10.1038/nature10166 21720365 PMC3163504

