# Supplementary figures and images for: DART: Denoising Algorithm based on Relevance network Topology improves molecular pathway activity inference
Source: BMC Bioinformatics. 2011 Oct 19;12:403. doi: 10.1186/1471-2105-12-403 (PMC3228554; doi:10.1186/1471-2105-12-403)

A)

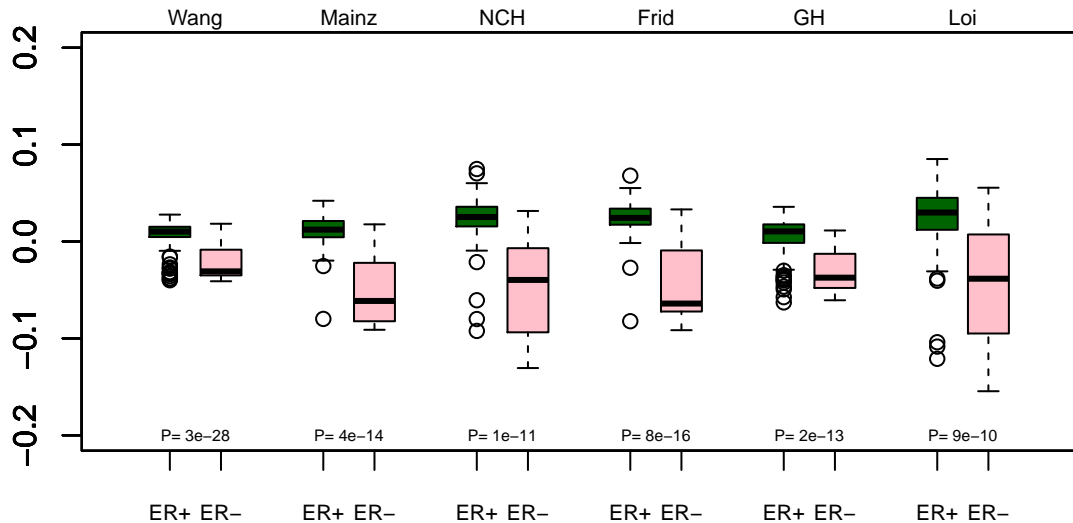

Supplement: Additional file 5 — DART ESR1 module in breast cancer. Boxplots comparing predicted pathway activities of the Doane ESR1 signature in ER+ versus ER- tumours in the six different breast cancer cohorts. P-values from a t-test are given. [file 1471-2105-12-403-S5.PDF]
